# Immunological In Vitro Assay for Quantification of Adjuvanted Allergoids

**DOI:** 10.1111/all.16543

**Published:** 2025-03-31

**Authors:** S. Schlünder, J. Echternach, D. Bartel, V. Mahler, M. D. Mühlebach, F. Führer

**Affiliations:** ^1^ Veterinary Medicine Division Paul‐Ehrlich‐Institut Langen Germany; ^2^ Allergology Division Paul‐Ehrlich‐Institut Langen Germany

**Keywords:** Adjuvanted immunotherapy products, Allergoid content assay, batch quality control, grass pollen allergen immunotherapy (AIT), regulatory framework

## Abstract

**Background:**

Many IgE‐mediated allergic disorders can be treated with allergen immunotherapy (AIT). In order to improve safety and efficacy, some AIT products contain allergen extracts which are chemically cross‐linked to generate allergoids and are adsorbed to aluminum hydroxide adjuvant. The modification and adsorption impair accessibility of the protein and quantification of the allergoid content.

**Methods:**

An ELISA‐like assay to quantify the allergoid content in adjuvanted grass pollen allergoid AIT products (from here on called: AIT drug products; AIT‐DPs) was developed using a fluorescence detection system. The high density of the aluminum hydroxide particles enabled pelleting the antigen complexes by centrifugation. Rabbit anti‐grass pollen allergoid sera or a mouse anti‐Phl p 5 monoclonal antibody (mAb) was used as the primary antibody. Protein content of the samples was quantified by nitrogen analysis.

**Results:**

High specificity of the primary antibodies was confirmed by isoelectric focusing, gel‐electrophoresis, and immunoblotting. Performance of the allergoid content assay was demonstrated in grass pollen AIT‐DPs with high specificity and low/absent cross‐reactivity with tree pollen or mite AIT‐DPs. It was used to confirm batch‐to‐batch consistency and allergoid content of distinct grass pollen AIT‐DPs. Overall, in relation to their total protein content, the allergoid content ranged between 0.8 and 2.1 relative to an in‐house reference for all grass pollen AIT‐DPs, whereas use of mAb revealed product‐specific differences in the Phl p 5 amount. Additionally, the assay detects product alteration by heat stress.

**Conclusion:**

The described assay is suitable to quantify the allergoid content and quality of allergoids in complex with aluminum hydroxide. It is suitable for animal‐free final product testing in vitro, for example, for batch release to ensure the quality of AIT‐DPs.

## Graphical abstract

An assay was developed to measure the allergoid content in adjuvanted grass allergoid AIT drug product (AIT‐DPs) at the finished product level. Both polyclonal rabbit sera or a mouse anti‐Phl p 5 mAb can be used, allowing differential product analysis. All four tested grass pollen AIT‐DPs of different manufacturers could be quantified using a defined reference, demonstrating broad applicability of the assay. ACA, allergoid content assay; AIT, allergen immunotherapy; AIT‐DP, adjuvanted allergoid AIT drug product; Al(OH)_3_, aluminum hydroxide; FITC, fluorescein isothiocyanate; mAb, monoclonal antibody; Phl p 5, Group 5 allergen *Phleum pratense* ; RAC, relative allergoid content.

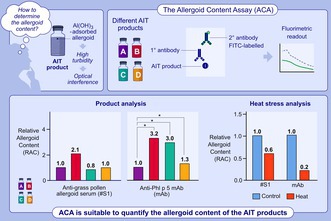

**Abbreviations**
ACA: Allergoid Content Assay
AIT: Allergen immunotherapy
AIT‐DP: Adjuvanted allergoid AIT drug product
AU: Arbitrary fluorescence units
BSA: Bovine serum albumin
DAFIA: Direct Alhydrogel Formulation Immunoassay
e.g.: *exempli gratia*
ELISA: Enzyme‐linked immunosorbent assay
EMA: European Medicines Agency
FITC: Fluorescein isothiocyanate
h: Hour
i.e.: *id est*
IEF: Isoelectric focusing
IgG: Immunoglobulin G
IHR: In‐house reference
kDa: Kilo Dalton
LoD: Limit of detection
mAb: Monoclonal antibody
min: Minute
nm: Nanometer
PBS‐T: Phosphate‐buffered saline containing 0.05% Tween‐20
*Ph. Eur*.: *European Pharmacopeia*
Phl p 5: Group 5 allergen *Phleum pratense*

pI: Isoelectric point
RAC: Relative allergoid content
RT: Room temperature
SD: Standard deviation
SDS‐PAGE: Sodium Dodecyl Sulfate Polyacrylamide Gel Electrophoresis
μg: Microgram
μL: Microliter

## 1 Introduction

The most common triggers of type I allergy are allergens from birch and grass pollen and house dust mites. Disease modifying treatment is performed by allergen immunotherapy (AIT) through repeated administration of the causative allergen [1, 2, 3]. AIT can achieve (partial) immunological tolerance and attenuation of the pathologic clinical immune response to the causative allergen [1, 2, 3]. However, AIT bears the risk of allergic or anaphylactic adverse effects [3]. In order to reduce allergenicity in some AIT products, allergens are chemically modified by cross‐linking, that is, by incubation of native allergen extracts with aldehydes (formaldehyde or glutaraldehyde), to generate high‐molecular‐weight allergoids [4, 5]. The aldehydes react with primary amines of the polypeptide chains of native allergens, which results in the formation of intra‐ and intermolecular cross‐links [3]. To enhance the immunogenicity of allergoids and native allergen extracts, they are adsorbed to aluminum hydroxide (Al(OH)_3_) adjuvant [6, 7], thereby generating allergoid/allergen‐aluminum nano‐ or microparticles [8]. Currently, several distinct aluminum hydroxide‐adjuvanted allergoid medicinal products for AIT of grass pollen allergy (from here on called: grass pollen AIT‐DPs^1^) are available on the German market and labeled in arbitrary units: Allergovit (10,000 TE/mL), Purethal (20,000 AUM/mL), Depigoid (1000 DPP/mL), Depigoid XT (1000 DPP/mL), Clustoid (10,000 TU/mL) and Roxoid (10,000 TU/mL). These grass pollen AIT‐DPs are composed of a mixture of pollen extracts of 5–10 species of the *Poaceae* family and *Pooideae* subfamily. The native pre‐manufacturing stage of modified allergoids is derived from natural source materials. Therefore, these products are complex mixtures of allergenic and non‐allergenic proteins and glycoproteins as well as other non‐protein components [4, 9]. Phl p 5 is a major allergen from Timothy grass ( *Phleum pratense* ). As Phl p 5 homologous are present in all grass pollen species, it is a key marker allergen within mixtures of different grass pollen extracts [10, 11, 12]. Due to the complexity of AIT‐DPs and the variability of the natural source materials, allergen products are subject to official experimental batch release testing according to section 32 of the German Medicinal Products Act [13]. The *European Pharmacopeia (Ph. Eur.)* requires that quality control of allergen products has to be performed at the latest possible step of the manufacturing process [14]. For allergoid products, determination of total allergenic activity is performed on the level of native extracts, as chemical modification impedes the IgE inhibition assay. Furthermore, according to the European Medicines Agency (EMA) “Guideline On Allergen Products: Production And Quality Issues” for modified allergens (e.g., chemically modified allergoids or conjugates), antibody‐based assays or other appropriate test methods have to be established to identify the relevant allergens in the modified form for characterization and control of the active substance in products [4, 15]. The adsorption to Al(OH)_3_ results in precipitation of the allergoid/allergen and turbidity of the AIT product. These product properties and the chemical modification of the allergen have so far prevented application of analytical methods such as ELISA for direct allergoid quantification in the final AIT‐DP as a measure for product potency [4, 16, 17]. Nonetheless, suitable methods for quality control are needed to ensure quality, safety and efficacy of AIT‐DP, including antigen content [18]. To fulfill the requirements of the EMA “Guideline On Allergen Products”, an Allergoid Content Assay (ACA) for the direct quantification in aluminum hydroxide‐adsorbed grass pollen AIT‐DPs is described here. Applicability of such an assay (“Direct Alhydrogel Formulation Immunoassay” (DAFIA) [16]) to determine antigen content in aluminum precipitate‐containing formulations was demonstrated previously for antigen quantification of an alum‐adjuvanted vaccine. Here we report the development of a simple, robust and rapid method for the quantitative determination of the allergoid content in the finished drug product.

## 2 Material and Methods

Detailed description of the used standard methods (Isoelectric focusing (IEF), Sodium Dodecyl Sulfate Polyacrylamide Gel Electrophoresis (SDS‐PAGE) and immunoblotting) as well as the used antibodies and desorption protocol, and samples are provided in Appendix S1.

### 2.1 Allergoid Content Assay (ACA)

The assay has been modified and adapted from a published method for the determination of malaria antigen formulated on Alhydrogel [16]. Grass pollen AIT‐DPs and in‐house reference (IHR) were pre‐diluted in phosphate‐buffered saline containing 0.05% Tween‐20 (PBS‐T) to achieve a defined equal protein content (6 μg protein/well). Al(OH)_3_ control samples without antigen (Alhydrogel adjuvant 2%, InvivoGen, Toulouse, France) were pre‐diluted in PBS‐T to achieve a similar aluminum content (20 μg Al(OH)_3_/well). Further, two‐fold serial dilutions were prepared with PBS‐T in triplicates in black 96‐well round‐bottom plates (200 μL/well) (BRAND Merck Darmstadt, Germany). Samples were pelleted by centrifugation at 1000 × *g* for 4 min at RT. Subsequently, the supernatant was removed and replaced by 200 μL PBS‐T by using a microplate washer (Tecan, Crailsheim, Germany); resuspension of pellets due to the operation of the microplate washer was sufficient. Determination of the amount of free allergoid not bound to the adjuvant (here: in the supernatants) is part of the standard quality control of AIT products and accounts for less than 10% of total protein. Thus, the loss of antigen during the ACA is not relevant. Additional resuspension was not necessary, evaluated during assay development. This process was defined as one washing step; three washing steps are defined as one washing cycle. After one washing cycle, the plates were blocked with 200 μL 2% BSA (Carl Roth, Karlsruhe, Germany) in PBS‐T, to abolish unspecific binding of the antibodies to the surface of the aluminum particles or the wells of the microplate. All incubation steps were performed on a microplate shaker with gentle rocking at room temperature (RT) for 1 h and followed by a washing cycle. 100 μL of primary antibodies (rabbit anti‐grass pollen allergoid sera (1:100) or mouse anti‐Phl p 5 monoclonal antibody (mAb) 1D11 (InBio, Cardiff, UK) (1:1000)) in blocking buffer was added, incubated, and followed by a washing cycle. 100 μL of secondary antibody (AffiniPure Goat Anti‐Rabbit IgG, FITC‐labeled (Jackson ImmunoResearch Laboratories, Cambridgeshire, UK) (1:100) or AffiniPure Goat Anti‐Mouse IgG, FITC‐labeled (Jackson ImmunoResearch Laboratories) (1:100)) in blocking buffer were added and incubated in the dark. Following a final washing cycle, pellets were resuspended in 100 μL PBS‐T, and fluorescence was measured by a microplate reader (Tecan Spark, Tecan) at 485 nm excitation/535 nm emission in the “automatic gain” modus. To allow calculation of the relative allergoid concentration (RAC), the in‐house reference was measured on each microtiter plate.

## 3 Results

### 3.1 Characterization of Primary Antibodies

**FIGURE 1:**
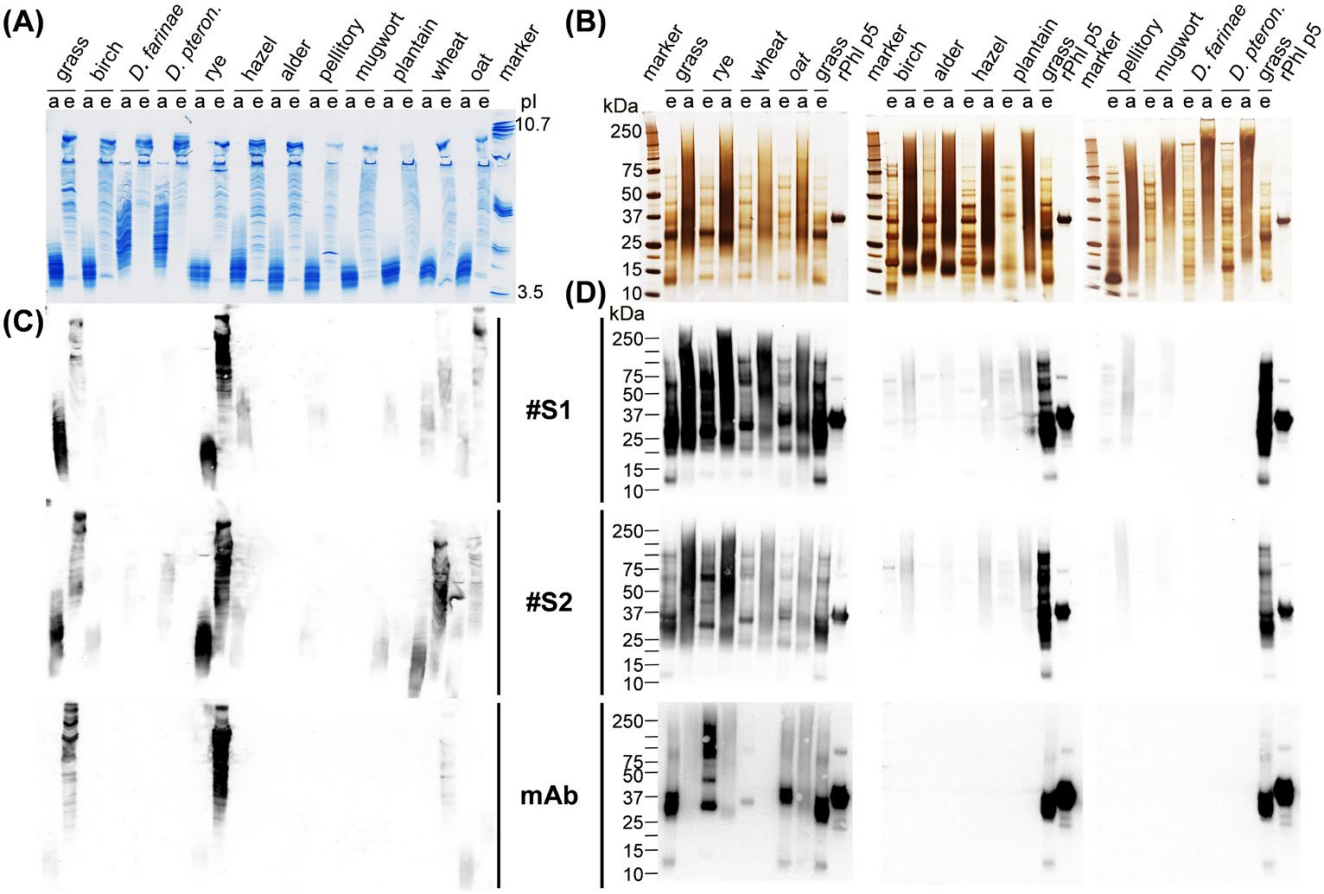
Specificity determination of primary antibodies. Representative results from three independent assays testing non‐adjuvanted allergen extracts (e) and non‐adjuvanted allergoids (a) from various allergen sources. (A) IEF gel after Coomassie staining. (B) SDS‐PAGE after silver staining. (C) and (D) immunoblotting with rabbit anti‐grass pollen allergoid serum 1 (#S1), rabbit anti‐grass pollen allergoid serum 2 (#S2), anti‐Phl p 5 mAb (mAb).

The specificity of polyclonal anti‐grass pollen allergoid sera of two rabbits (#S1 and #S2) as well as of an anti‐Phl p 5 mAb was determined. First, their binding to native allergen extracts and cross‐linked allergoids, both non‐adjuvanted precursors of AIT‐DPs, from different species and manufacturers were analyzed by IEF (Figure 1A and Figure S1A) and SDS‐PAGE (Figure 1B and Figure S1B). The IEF results show defined and discrete protein patterns for the native allergen extracts (Figure 1A and Figure S1A). A strong shift of the isoelectric point (pI) to acidic pH values became apparent for all cross‐linked allergoids in comparison to the respective allergen extracts, accompanied by a more diffuse band pattern. Moreover, the impact of cross‐linking became also visible in SDS‐PAGE (Figure 1B and Figure S1B). No discrete protein bands were observed for any tested allergoid, in contrast to the native allergen precursors. Furthermore, there was an overall shift of molecular weights of allergoids toward higher apparent mass. The results of both experiments indicate the formation of high‐molecular protein aggregates by chemical cross‐linking. As expected, the allergen extracts contained a variety of proteins between 10 and 200 kDa, reflecting the high complexity of the natural source material. Second, the IEF immunoblotting with sera #S1, #S2, or anti‐Phl p 5 mAb (Figure 1C and Figure S1C) revealed specific binding of the sera to allergens or allergoids within the homologous group of grass pollen, that is, grass, rye, wheat, and oat. For sera #S1 and #S2, faint background binding to other allergoids was observed. SDS‐PAGE followed by Western immunoblotting analysis revealed the highly specific detection of allergens of different manufacturers (A–D) (Figure 1D and Figure S1D). After an exposure time of up to 30 s, faint detection of other allergoids was discernible. Anti‐Phl p 5 mAb showed only binding to samples of the grass pollen group (Figure 1D) confirming the high specificity of this mAb and the different molecular weights of Phl p 5 homologs within the *Poaceae* family.

### 3.2 Development of the Allergoid Content Assay (ACA)

**FIGURE 2:**
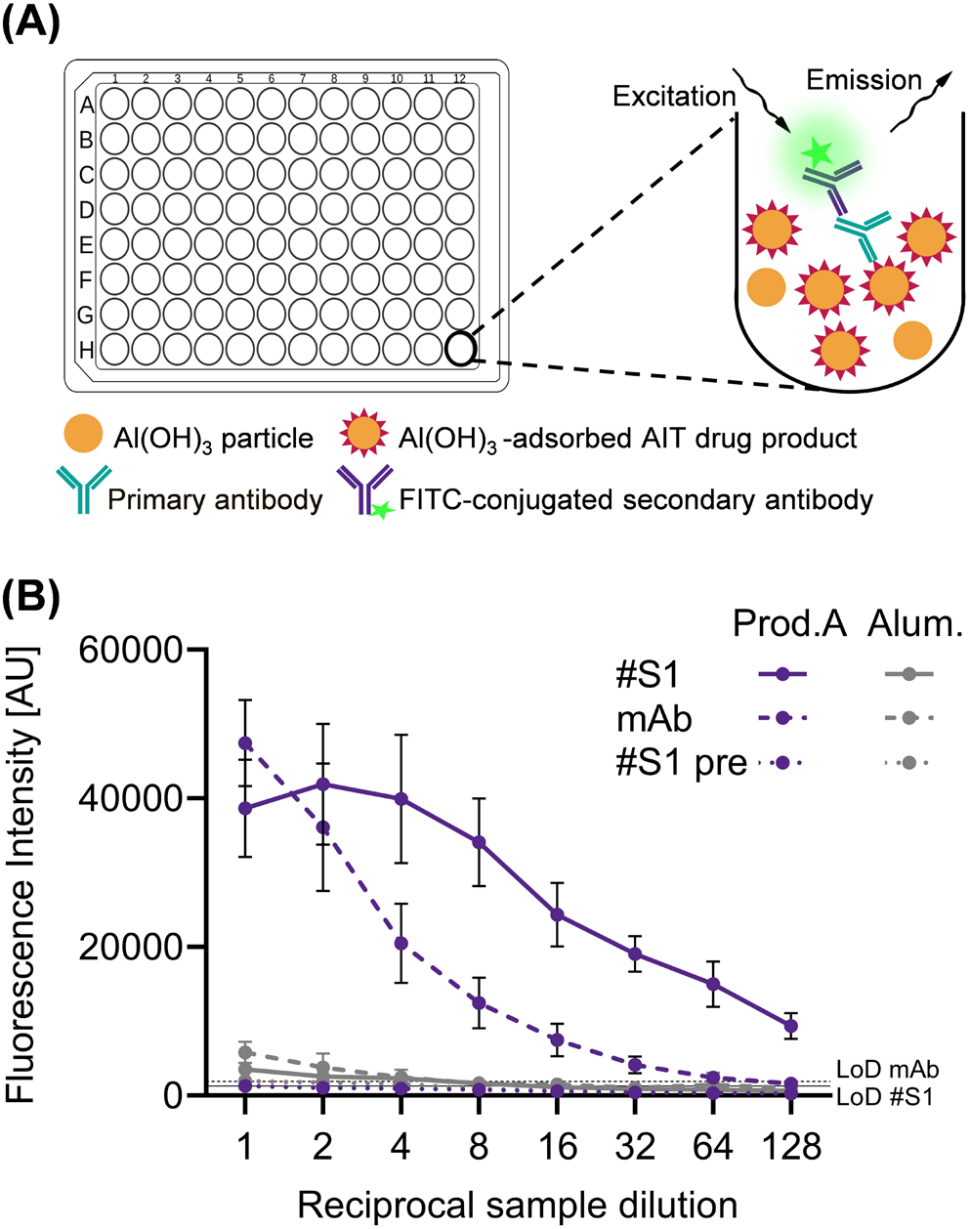
Allergoid Content Assay (ACA). (A) Schematic illustration of ACA. Individual components of the assay are depicted. (B) Proof of Concept of ACA and determination of sensitivity. Mean signals from three independent assays performed in triplicates of testing adjuvanted grass pollen allergoid AIT product A (purple) or an aluminum hydroxide control (gray) with anti‐grass pollen allergoid serum 1 (#S1, continuous line), anti‐Phl p 5 mAb (mAb, dashed line); or pre‐immune serum (#S1 pre, dotted line). AU, arbitrary fluorescence units. Error bars indicate standard deviation SD. Limit of detection (LoD) displayed for anti‐grass pollen allergoid serum 1 (#S1, continuous line) and anti‐Phl p 5 mAb (mAb, dashed line).

In order to quantify allergoids adsorbed to aluminum hydroxide particles directly in AIT‐DPs, a fluorescence‐based assay in microtiter plate format was developed. For this purpose, sera or mAbs have to be identified that are able to bind with high affinity to the allergoids irrespective of the adsorption to aluminum hydroxide particles. Due to the high turbidity of the medicinal products, a direct colorimetric readout is hardly feasible. However, a fluorometric readout, employing a fluorophore‐conjugated secondary antibody, was proposed to overcome such optical interference [16]. In this assay format, the macroscopic nature of the aluminum hydroxide particles is used for co‐pelleting the adsorbed antigen by centrifugation. After centrifugation, the supernatant can be removed and replaced by fresh buffer so that the pelleted sample material can be washed. Hence, there is no need for antigen adsorption to additional stationary matrices like plates, as for example, required for ELISA (Figure 2A). Parameters to be optimized during development were protein content of the samples, blocking agent and its concentration, dilution of primary and secondary antibodies, and the mode of washing procedure. General proof of concept of the ACA was provided by testing adjuvanted grass pollen AIT drug product A with either serum #S1 or anti‐Phl p 5 mAb as primary antibodies. Al(OH)_3_ control samples and pre‐immune serum of the rabbit used to generate serum #S1 served as negative and specificity controls, respectively. Serial dilutions of a grass pollen AIT‐DP A (6 μg allergoid adjuvanted with approx. 20 μg Al(OH)_3_) and an aluminum hydroxide control (20 μg Al(OH)_3_ without antigen) were analyzed, and respective data are shown in Figure 2B. High signal intensities and a dose‐dependent decreasing signal are shown for grass pollen AIT‐DP A with serum #S1 as well as for the mAb. The low signal for the Al(OH)_3_ control (approx. 10‐fold less than the grass pollen AIT‐DP) with both tested primary antibodies demonstrates considerably low unspecific binding of the primary antibodies to the Al(OH)_3_ particles. Furthermore, for sensitivity validation, the signals of the aluminum control (dilution 1:128) detected with serum #S1 and mAb were used for determining the limit of detection (LoD) of the developed ACA (Figure 2B). With the pre‐immune serum #S1, even lower relative signals (approx. 30‐fold less than the grass pollen AIT‐DP) were detected. These results confirm the suitability of the grass pollen ACA to determine the grass pollen allergoid content with high sensitivity in Al(OH)_3_‐formulated grass pollen AIT‐DPs.

**FIGURE 3:**
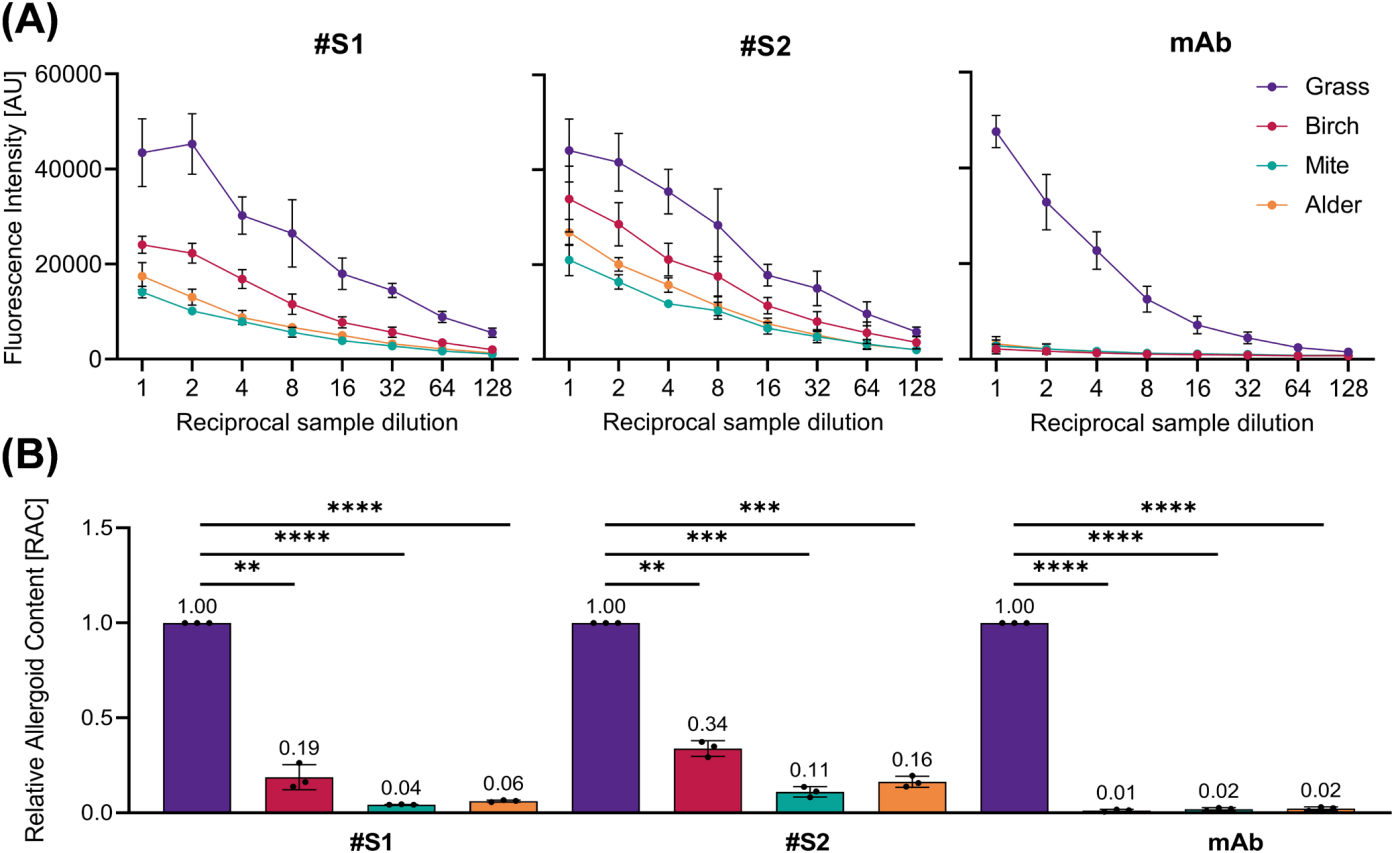
Specificity determination of ACA. (A) Means from three independent assays performed in triplicates of testing adjuvanted allergoid AIT products (purple, grass; red, birch; green, mite; orange, alder) with anti‐grass pollen allergoid serum 1 (#S1), anti‐grass pollen allergoid serum 2 (#S2) or anti‐Phl p 5 mAb (mAb). AU, arbitrary fluorescence units; error bars indicate standard deviation SD. (B) Relative allergoid content for adjuvanted allergoid AIT products (purple, grass; red, birch; green, mite; orange, alder). Dots depict RAC of individual experiments; error bars depict SD; numbers above bars indicate mean RAC. Asterisks depict *p* values levels of statistical significance (****, < 0.0001, extremely significant; ***, 0.0001–0.001, extremely significant; **, 0.001–0.01, very significant).

Specificity of grass pollen ACA was assessed by testing AIT‐DPs of different species (grass, birch, mite and alder) with #S1, #S2, or anti‐Phl p 5 mAb (Figure 3). High signals above 40,000 arbitrary fluorescence units (AU) were detected with all three tested primary antibodies for the grass pollen AIT‐DP (Figure 3A). For the other AIT‐DPs (birch, mite and alder), lower signals were observed using sera #S1 and #S2 as primary antibodies. In the case of the birch AIT‐DP (24,096 AU #S1; 33,804 AU #S2), signals of the undiluted sample were lower than the signals of the 1:4 diluted grass pollen AIT‐DP using both sera, indicating at least a four‐fold higher specificity. Signals obtained with the alder (17,480 AU #S1; 26,779 AU #S2) and mite (14,127 AU #S1; 20,938 AU #S2) AIT‐DPs were even lower (Figure 3A). Using the mAb as primary antibody, differences in signal intensity became considerably more pronounced, with 47,629 AU for the grass pollen AIT‐DP compared to 3242 AU for the tested alder AIT‐DP. Even lower signals were obtained for the birch (2147 AU) and mite (2839 AU) AIT‐DPs. For further evaluation, the relative allergoid content (RAC) was calculated with CombiStats for all tested AIT‐DPs (Figure 3B) in relation to the defined in‐house reference (IHR) which is included in each microtiter plate. This IHR was assigned an arbitrary value of 1 RAC. Calculated RACs using sera #S1 or #S2 are 0.19 and 0.34 for the birch AIT‐DP, respectively. In the case of alder and mite products, the RACs are below 0.16. As expected, the calculated RAC with the mAb as primary antibody was almost zero (below or equal to 0.02) for all non‐grass pollen AIT‐DPs (Figure 3B). Significance analysis of calculated RAC values (Figure 3B) confirmed the high specificity of the three tested primary antibodies to grass pollen allergoids in comparison to allergoids in other AIT‐DPs (birch, mite and alder) with a high significance (at least *p* value of 0.001–0.01).

### 3.3 Assessment of Batch‐To‐Batch Consistency Using the ACA

**FIGURE 4:**
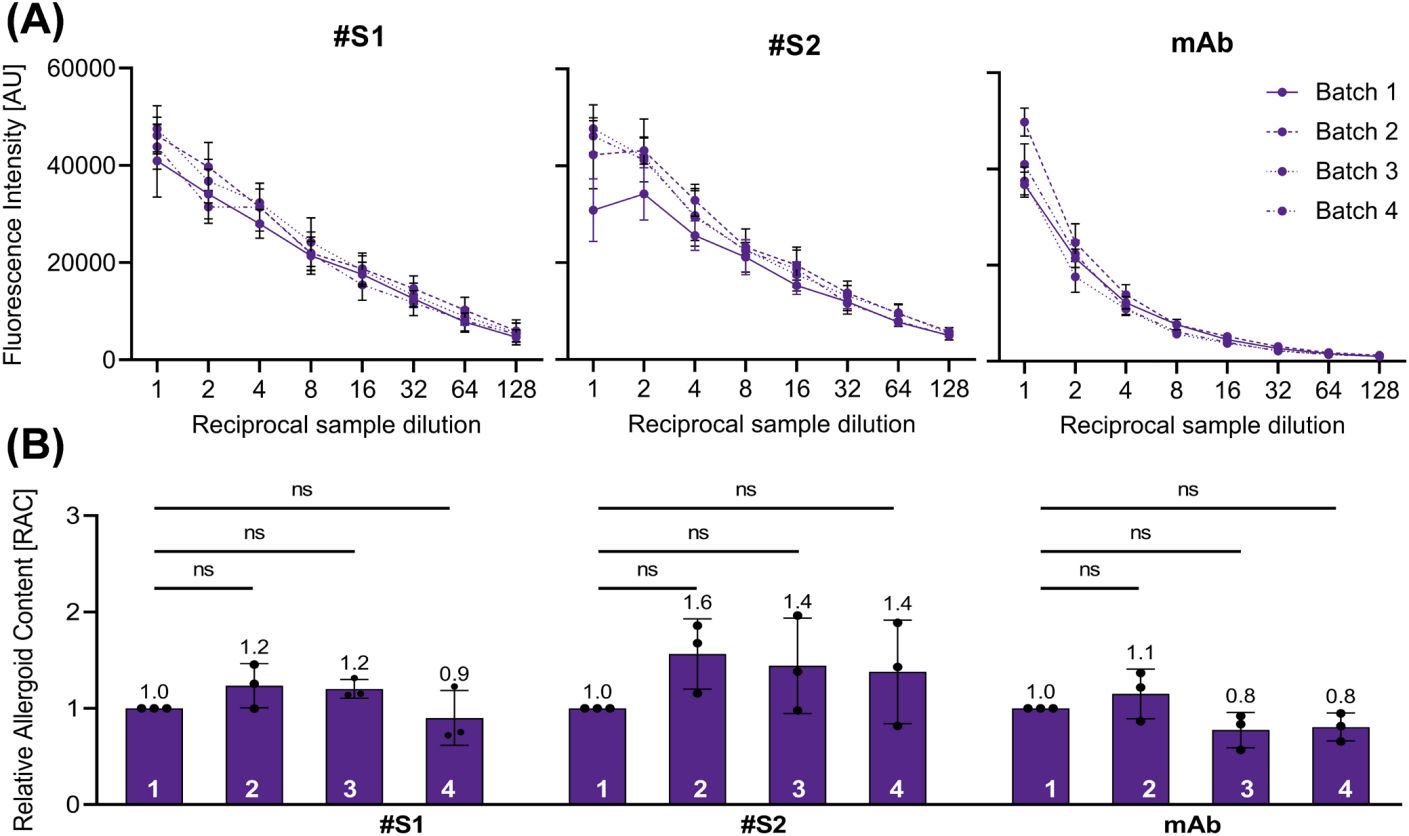
Assessment of batch‐to‐batch consistency. Means from three independent experiments each performed in triplicates testing four different batches of an adjuvanted grass pollen allergoid AIT product A with (A) anti‐grass pollen allergoid serum 1 (#S1), anti‐grass pollen allergoid serum 2 (#S2), or anti‐Phl p 5 mAb (mAb). AU, arbitrary fluorescence units, error bars depict standard deviation SD. (B) Relative allergoid content of four different batches (1–4 as indicated) of adjuvanted grass pollen allergoid AIT product A. Dots depict RAC of individual experiments; error bars depict SD; numbers above bars indicate mean RAC. Asterisks depict *p* values levels of statistical significance (ns, ≥ 0.05, not significant).

Applicability to use ACA for batch‐to‐batch consistency testing was evaluated by analyzing four different batches of grass pollen AIT‐DP A with sera #S1, #S2, and anti‐Phl p 5 mAb (Figure 4). Low variability and high consistency of ACA is shown by low standard deviation (SD) between the three independent experiments (Figure 4A). RAC of the different batches was calculated as described above. The allergoid content of the four tested batches using serum #S1 for the assay yielded 0.9–1.2 RAC. Similar variation (0.8–1.1 RAC) resulted with the mAb as the primary antibody (Figure 4B). Higher apparent content differences between the tested batches were calculated using serum #S2 (1.0–1.6 RAC). Thus, for serum #S1 and the mAb, the relative variance of calculated RAC ranged between 8% and 32%. Due to the lower standard deviations of the experiments and a higher specificity with sera #S1, only this serum was used in all further analyses. Significance analysis of calculated RAC values (Figure 4B) revealed no significant (≥ 0.05 *p* value) differences between the four different batches (1–4 as indicated) of adjuvanted grass pollen allergoid AIT product A for all three tested primary antibodies. These results indicate batch‐to‐batch variation in a range, as expected for products of biological origin, and fulfilling the recommendations of total allergenic activity by the European Pharmacopeia employing the ACA to determine the allergoid content of a grass pollen AIT‐DP of different batches of one manufacturer.

### 3.4 Determination of Allergoid Content of Grass Pollen AIT Drug Products From Different Manufacturers

**FIGURE 5:**
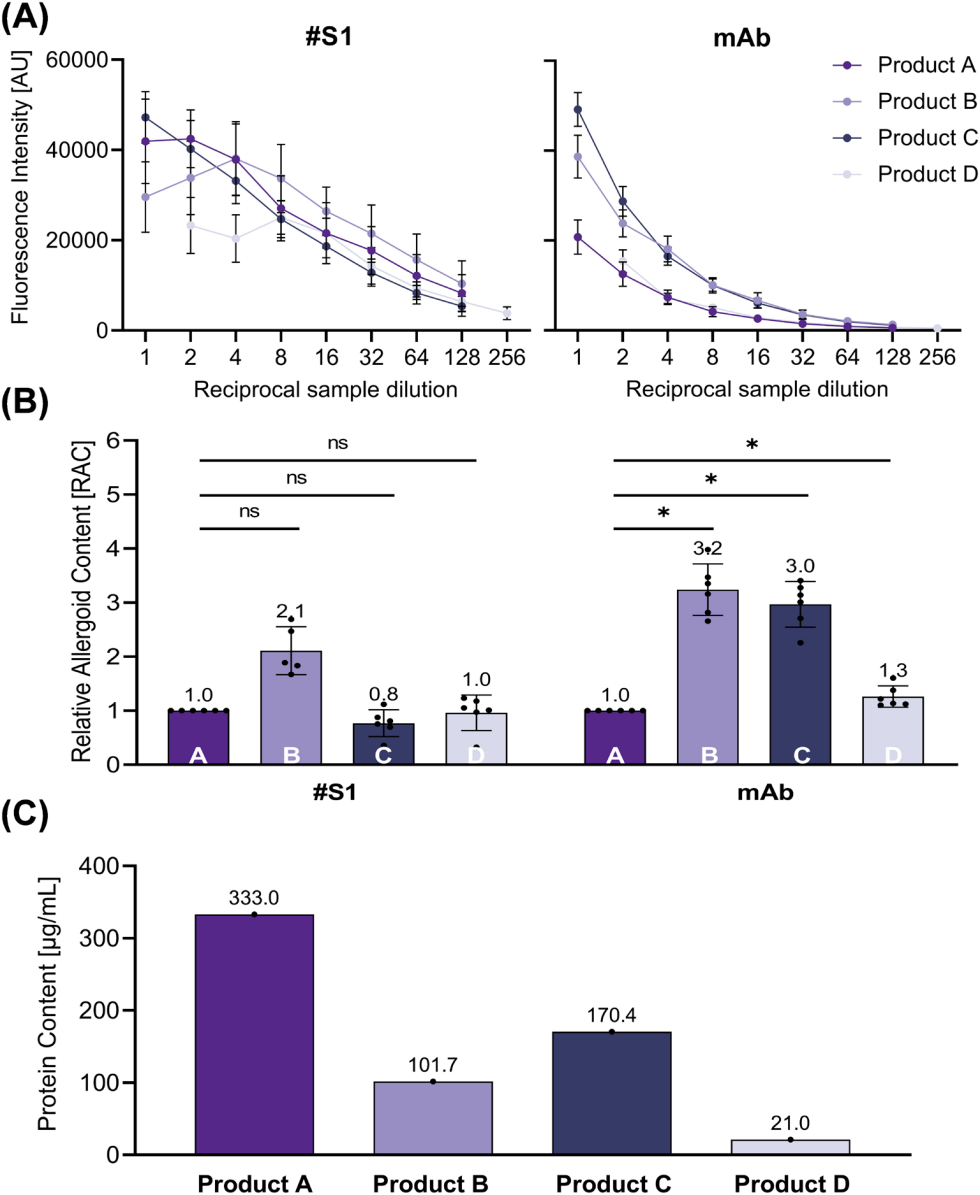
Product specific relative allergoid content of adjuvanted grass pollen allergoid AIT products of different manufacturers. (A) Means from at least five independent experiments each performed in triplicates of testing one batch of adjuvanted grass pollen allergoid AIT products from different manufactures A–D as indicated with anti‐grass pollen allergoid serum 1 (#S1), or anti‐Phl p 5 mAb (mAb). AU, arbitrary fluorescence units error, bars depict standard deviation SD (B) Relative allergoid content of different adjuvanted grass pollen allergoid AIT products. Dots depict RAC of individual experiments; error bars depict SD; numbers above bars indicate mean RAC. Asterisks depict *p* values levels of statistical significance (*, 0.01–0.05, significant; ns, ≥ 0.05, not significant). (C) Protein content of adjuvanted grass pollen allergoid AIT products A–D used for analysis in (A) and (B). Dots depict protein content of one experiment (performed in duplicates); numbers above bars indicate protein content.

The allergoid content of four different grass pollen AIT‐DPs from different manufacturers (one batch each A to D) was investigated by using ACA with serum #S1 or anti‐Phl p 5 mAb as primary antibodies (Figure 5). Sample preparation was identical for all samples, with an exception for product D. Due to its lower protein content, the initial amount of protein in the dilution series was set at 3 μg (All others: 6 μg). The dose response curves for all products are shown in Figure 5A. Signals at 1:8 dilution of the assays generated with serum #S1 yielded lower signals for products C and D (24,653 and 25,019 AU, respectively) and higher signals for product B (33,655 AU) in comparison to product A (27,076 AU). Furthermore, signals of products B and D show at the first three sample dilutions no dose‐dependent decrease. Both products plateau in the first three dilutions, followed by a dose‐dependent decrease of signals at further product dilutions, enabling parallel regressions of the dose–response curves for calculation of RAC only by excluding the low dilutions for calculation. Detection of Phl p 5 using the mAb enables independent investigation of the analyzed four different AIT‐DPs, unbiased from a potential influence of the allergoid choice used to produce the polyclonal rabbit sera which represents a potential bias/confounder in favor of the respective product. It allows direct comparison of Phl p 5 allergoid content among the AIT‐DPs. Dose response curves obtained with the mAb are also more reproducible in comparison to those with serum #S1 indicated by generally smaller SDs (Figure 5A). For comparison of products, RAC of product B, C, and D were calculated in relation to the IHR (i.e., Batch 1 of product A), as described above (Figure 5B). By using serum #S1, detected allergoid contents of product C (0.8 RAC) and D (1.0 RAC) are in the same range as product A (1.0 RAC). Remarkably, product B (2.1 RAC) revealed a more than two‐fold higher RAC. Significance analysis (Figure 5B) revealed no significant (≥ 0.05 *p* value) difference between the four adjuvanted grass pollen allergoid AIT products from different manufacturers A–D by using anti‐grass pollen allergoid serum #S1 as primary antibody. Calculation of RAC according to the Phl p 5 content using mAb for detection revealed three‐fold higher contents of products B (3.2 RAC) and C (3.0 RAC) compared to products A (1.0 RAC) or D (1.3 RAC) and a statistically significant difference (*p* value 0.01–0.05) between the four tested products. In summary, using the ACA in combination with an IHR allows determining allergoid content and calculating the relative allergoid content for all tested product batches. The protein content of the analyzed products was determined by the Kjeldahl method (Figure 5C).

### 3.5 Analysis of Product Stability Using ACA

**FIGURE 6:**
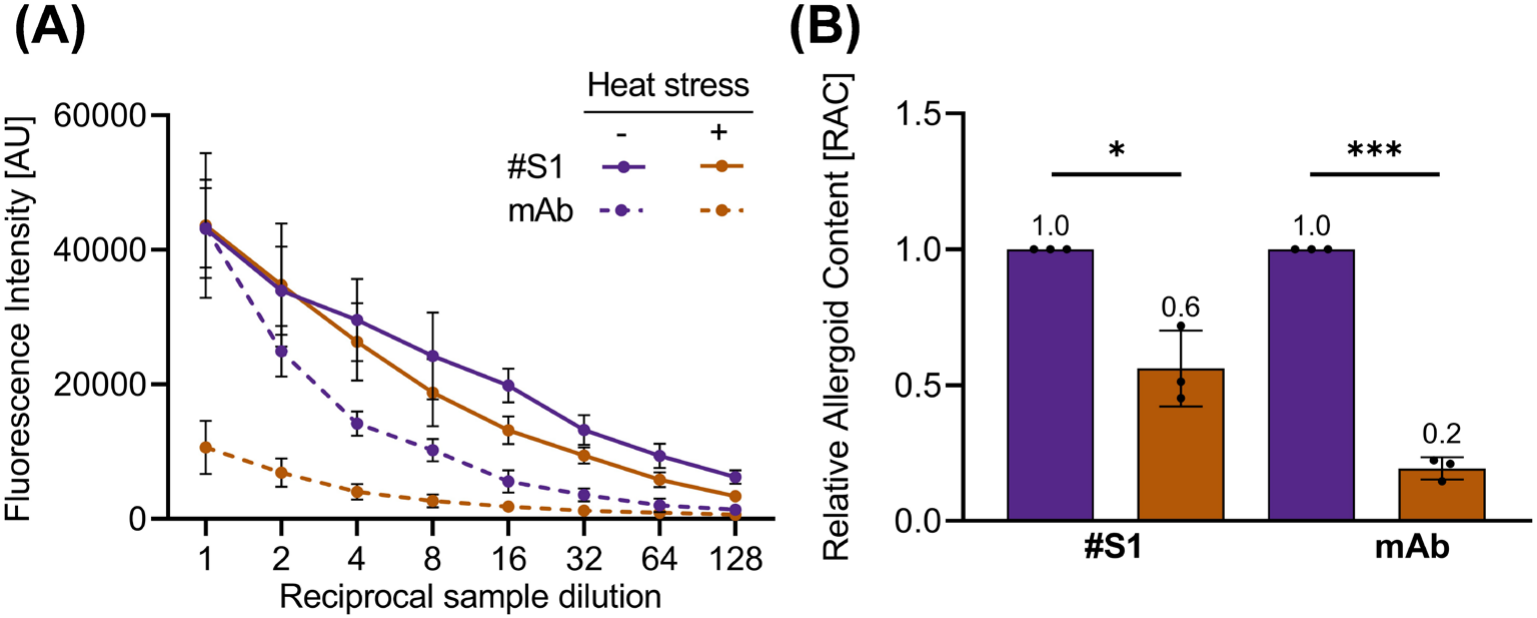
Identification of ACA as stability indicating assay. (A) Means from three independent experiments each performed in triplicates of testing adjuvanted grass pollen allergoid AIT product A before (purple) or after heat stress procedure (brown), with anti‐grass pollen allergoid serum 1 (#S1, continuous line) or anti‐Phl p 5 mAb (mAb, dashed line). AU, arbitrary fluorescence units error, bars depict standard deviation SD (B) Relative allergoid content of adjuvanted grass pollen allergoid AIT product A before and after heat stress procedure. Dots depict RAC of individual experiments; error bars depict SD; numbers above bars indicate mean RAC. Asterisks depict *p* values levels of statistical significance (***, 0.0001–0.001, extremely significant; *, 0.01–0.05, significant).

To assess the potential of the grass pollen ACA to detect alterations of batches and to monitor product stability, Batch 1 of grass pollen AIT drug product A was subjected to forced degradation by heating. This stressed sample was subsequently investigated by ACA in comparison to an untreated control sample. Both samples were analyzed employing serum #S1, or the mAb as primary antibodies (Figure 6). Dose response curves in ACA of heat‐treated and untreated control samples with serum #S1 were distinguishable (Figure 6A). The signal intensity of the treated sample (18,779 AU, 1:8 dilution) is roughly two‐fold lower in comparison with the untreated sample (24,241 AU, 1:8 dilution). When anti‐Phl p 5 mAb was used as the primary antibody, these differences became even more prominent (treated sample 2672 AU, 1:8 dilution; untreated sample 10,246 AU, 1:8 dilution). Only approx. 28% of the signal intensity of the untreated sample remained after heat treatment. The RAC of the treated sample was calculated using the untreated sample as a reference (Figure 6B) and revealed a significant reduction by 50% or 80% when using serum #S1 (*p* value 0.01–0.05; significant) or mAb (*p* value 0.0001–0.001; extremely significant), respectively. Thus, the ACA recognized changes in the products after heat stress of a grass pollen AIT‐DP.

## 4 Discussion

In this study, an in vitro immunological test system to quantify aluminum hydroxide‐adsorbed grass pollen allergoid in AIT drug products^1^ was established to determine product potency according to the EMA guideline [4, 15]. The presented grass pollen ACA has been newly developed based on a previously described direct Alhydrogel formulation immunoassay (DAFIA) for the determination of the antigen content in an aluminum hydroxide‐adjuvanted malaria vaccine [16]. While such applicability of the immunoassay was confirmed also for other vaccines, containing for example, diphtheria toxoid [19, 20], for AIT products comparable analyses had been lacking. For this purpose, primary antibodies are required to detect the adsorbed allergens or allergoids. Here, polyclonal sera of two immunized rabbits as well as a Phl p 5‐specific monoclonal antibody were used to cover a broad antigen and epitope reservoir, and to detect the major and highly cross‐reactive timothy grass pollen allergen, Phl p 5 [10, 11, 12]. All three primary antibodies revealed high specificity to corresponding non‐adjuvanted allergens or allergoids out of the homologous grass pollen group confirming their principal suitability for the intended immunoassay [15]. The non‐adjuvanted allergoids show the characteristic smear after SDS‐PAGE but clear band patterns when separated in IEF [4]. The observed high reactivity of sera and anti‐Phl p 5 mAb to pollen allergens of different species of the *Poaceae* family and *Pooideae* subfamily, that is, grass, rye, wheat, and oat, enabled testing of different adjuvanted grass pollen AIT drug products of various species compositions [21, 22]. With much lower intensity, also a reactivity of rabbit sera with non‐adjuvanted allergoids of mite, birch, or herbal pollen was observed (Figure 1). These might be caused by the formation of neoepitopes during the modification process, independent of allergen species, and is going to be investigated in a follow‐up study. After demonstrating high but conserved specificity of primary antibodies, proof of concept of the ACA for direct allergoid quantification was demonstrated for one specific grass pollen AIT‐DP. Before, only indirect methods had been available for quantification of allergoids after desorption from the aluminum adjuvant, as recommended for vaccines [23]. However, the desorption process is time‐consuming, laborious, hard to validate, and can alter the antigen structure [16, 17]. Our assay circumvents this critical step and enables direct and fast antigen quantification. After proof of concept, high specificity of primary antibodies for grass pollen allergens and allergoids was demonstrated to be indeed transferable to the ACA for grass pollen AIT‐DP content as indicated by low or no signals against other AIT‐DPs (from birch, mite or alder). AIT products are complex mixtures of allergens, co‐extracted non‐allergenic proteins, and other biological substances and therefore pose a considerable challenge for the implementation and performance of such an immunoassay. Nevertheless, the ACA showed overall high allergen specificity. The prominent differences in specificity between sera #S1 and #S2, and especially between both sera and anti‐Phl p 5 mAb were apparent. The detected variability between the two rabbit sera, cross‐reactivities, and a moderate background are well‐known drawbacks of generation of primary antibodies via animal immunization [24]. Binding and high specificity of anti‐Phl p 5 mAb to native grass pollen extracts was previously described and expected [22, 25]. The binding of anti‐Phl p 5 mAb to the specific Phl p 5 epitope [25] was found maintained despite cross‐linking in allergoids (grass, rye, wheat, and oat). Emphasizing the fact that the respective IgG Phl p 5 epitope is not altered by cross‐linking allows for the use of this anti‐Phl p 5 mAb for quantification of allergoids in the final product. Furthermore, the ACA is suitable for analyzing batch‐to‐batch consistency and comparability of grass pollen AIT‐DPs from different manufacturers by determination of their allergoid content. Analyzing the same protein amounts of the four grass pollen AIT‐DPs from different manufacturers available in the German market, up to two‐fold allergoid content was found. It should be noted that although the sera #S1 and #S2 were generated by using one allergoid batch of one manufacturer (product A), they perform rather product‐independent in the assay. Despite one batch of product A served as IHR, a selective preference of ACA for product A was not detectable. Neither product B nor its non‐adjuvanted allergoid precursor was used for immunization of the rabbits; however, product B achieved the highest signals and revealed a two‐fold higher detectable RAC in comparison to the three other products. Highest differences in RAC between all analyzed products were detected using the anti‐Phl p 5 mAb, showing up to three‐fold different content. RAC results using the anti‐Phl p 5 mAb are in the same order of magnitude as using the rabbit sera; the bias due to the immunization of the rabbits with one preparation has no major influence on the detection of all different preparations. ACA using the anti‐Phl p 5 mAb allows product‐independent analysis of one relevant antigen, whereas the use of rabbit sera allows detection of many different antigens with the disadvantage of a potential bias due to the immunization with one preparation. Finally, the evaluation of batch‐to‐batch consistency is possible with both. Up to now, no assessment of product potency of different AIT‐DPs was feasible. In contrast to AIT products, for vaccine products, potency and safety can be studied in suitable animal models assessing their immune reactions, as done for example, for diphtheria or rabies vaccines [26]. Nonetheless, tests in animals need to be replaced with in vitro assays in line with 3R principles [27] wherever possible [28]. To show immunogenicity of AIT products, animals are used, for example in preclinical trials. The established in vitro ACA to determine product potency is a complementary tool to reduce the use of animals. Interestingly, heat‐stressed material evoked significantly lower detection signals in the ACA, especially when using the mAb as primary antibody. Consequently, this assay can differentiate between well‐folded and heat‐degraded proteins and thereby integrity of the grass pollen AIT‐DP, a hallmark of product quality testing. However, some limitations of the study exist, that is, absolute quantification of allergoids is impossible in the absence of an international standard. In addition, direct correlation of allergoid content and allergenicity is not possible. Animal models to directly investigate allergenicity in vivo, however, may not be considered appropriate for routine use due to 3R principles and questionable transferability to the human setting.

Taken together, the ACA is a promising assay to identify and relatively quantify immunologically active (glutaraldehyde or formaldehyde) modified proteins of grass pollen AIT‐DPs and will be implemented to improve quality control testing of finished products, in addition to the determination of total allergenic activity at the level of native allergen extracts [15]. The ACA could also be further developed for other allergen sources. Especially, the use of well‐suited mAbs was demonstrated to gain less variable results with higher specificity and better sensitivity for product degradation. In addition, the mAb used opens the opportunity for sustainable assay standardization toward *Ph. Eur*. Compared to rabbit sera, which are inherently limited, the mAb can be produced forever, maintained, and distributed as a reagent for a potential future reference method.

## Author Contributions

Simone Schlünder performed all laboratory assays. Simone Schlünder, Johanna Echternach, Detlef Bartel, Michael D. Mühlebach and Frank Führer interpreted the data. Johanna Echternach, Detlef Bartel, Michael D. Mühlebach, and Frank Führer supervised the study. Simone Schlünder, Johanna Echternach, Vera Mahler, Michael D. Mühlebach, and Frank Führer wrote the paper. All authors reviewed the manuscript.

## Disclosure

The views expressed in this paper are the personal views of the author and may not be understood or quoted as being made on behalf of or reflecting the position of the respective national competent authority, the European Medicines Agency, or one of its committees or working parties.

## Conflicts of Interest

The authors declare no conflicts of interest.

## Supporting information


Figure S1.

Appendix S1.

## Data Availability

The data that support the findings of this study are available from the corresponding author upon reasonable request.

## Endnotes

^1^
Drug product: The form of the completed pharmaceutical product; here: adjuvanted allergoid AIT drug products (AIT‐DPs). In the entire manuscript the authors refer to adjuvanted allergoids when using the abbreviation AIT‐DP.

## References

[1] S. H. Arshad , “An Update on Allergen Immunotherapy,” Clinical Medicine (London, England) 16, no. 6 (2016): 584–587, 10.7861/clinmedicine.16-6-584. 27927826 PMC6297333

[2] U. C. Kucuksezer , C. Ozdemir , L. Cevhertas , I. Ogulur , M. Akdis , and C. A. Akdis , “Mechanisms of Allergen‐Specific Immunotherapy and Allergen Tolerance,” Allergology International 69, no. 4 (2020): 549–560, 10.1016/j.alit.2020.08.002. 32900655

[3] B. Heydenreich , I. Bellinghausen , L. Lund , et al., “Adjuvant Effects of Aluminium Hydroxide‐Adsorbed Allergens and Allergoids—Differences In Vivo and In Vitro,” Clinical and Experimental Immunology 176, no. 3 (2014): 310–319, 10.1111/cei.12294. 24528247 PMC4008974

[4] J. Zimmer , A. Bonertz , and S. Vieths , “Quality Requirements for Allergen Extracts and Allergoids for Allergen Immunotherapy,” Allergologia et Immunopathologia 45 (2017): 4–11, 10.1016/j.aller.2017.09.002. 29128092

[5] I. Ibarrola , M. L. Sanz , P. M. Gamboa , et al., “Biological Characterization of Glutaraldehyde‐Modified *Parietaria judaica* Pollen Extracts,” Clinical and Experimental Allergy 34, no. 2 (2004): 303–309, 10.1111/j.1365-2222.2004.01859.x. 14987312

[6] A. I. Joubert , M. Geppert , L. Johnson , et al., “Mechanisms of Particles in Sensitization, Effector Function and Therapy of Allergic Disease,” Frontiers in Immunology 11 (2020): 1334, 10.3389/fimmu.2020.01334. 32714326 PMC7344151

[7] A. R. Zoss , C. A. Koch , and R. S. Hirose , “Alum‐Ragweed Precipitate: Preparation and Clinical Investigation,” Journal of Allergy 8, no. 4 (1937): 329–335, 10.1016/S0021-8707(37)90140-0.

[8] X. Li , A. M. Aldayel , and Z. Cui , “Aluminum Hydroxide Nanoparticles Show a Stronger Vaccine Adjuvant Activity Than Traditional Aluminum Hydroxide Microparticles,” Journal of Controlled Release 173 (2014): 148–157, 10.1016/j.jconrel.2013.10.032. 24188959 PMC3918952

[9] I. J. Ansotegui , G. Melioli , G. W. Canonica , et al., “IgE Allergy Diagnostics and Other Relevant Tests in Allergy, a World Allergy Organization Position Paper,” World Allergy Organization Journal 13, no. 2 (2020): 100080, 10.1016/j.waojou.2019.100080. 32128023 PMC7044795

[10] K. Andersson and J. Lidholm , “Characteristics and Immunobiology of Grass Pollen Allergens,” International Archives of Allergy and Immunology 130, no. 2 (2003): 87–107, 10.1159/000069013. 12673063

[11] M. Hrabina , G. Peltre , R. van Ree , and P. Moingeon , “Grass pollen allergens,” Clinical and Experimental Allergy Reviews 8, no. 1 (2008): 7–11, 10.1111/j.1472-9733.2008.00126.x.

[12] I. Sander , C. Fleischer , U. Meurer , T. Brüning , and M. Raulf‐Heimsoth , “Allergen Content of Grass Pollen Preparations for Skin Prick Testing and Sublingual Immunotherapy,” Allergy 64, no. 10 (2009): 1486–1492, 10.1111/j.1398-9995.2009.02040.x. 19385952

[13] “Gesetz Über Den Verkehr Mit Arzneimitteln (Arzneimittelgesetz) in Der Fassung Der Bekanntmachung Vom 12. Dezember 2005 (BGBl. I S. 3394), Das Zuletzt Durch Artikel 7 Des Gesetzes Vom 27. März 2024 (BGBl. 2024 I Nr. 109) Geändert Worden Ist. [Medicinal Products Act (Arzneimittelgesetz–AMG) in the Version Published on 12 December 2005 (Federal Law Gazette [BGBl.]) Part I P. 3394, Last Amended by Article 7 of the Act of 27 March 2024 (Federal Law Gazette 2024 I No. 109)]”.

[14] EDQM , “Monograph on Allergen Products (01/2022:1063). European Pharmacopoeia 11.0”.

[15] European Medicines Agency. Committee for Medicinal Products for Human Use (CHMP) , “Guideline on Allergen Products: Production and Quality Issues (EMA/CHMP/BWP/304831/2007)”.

[16] D. Zhu , S. Huang , E. Gebregeorgis , et al., “Development of a Direct Alhydrogel Formulation Immunoassay (DAFIA),” Journal of Immunological Methods 344, no. 1 (2009): 73–78, 10.1016/j.jim.2009.03.005. 19328804 PMC2712229

[17] D. Laera , H. HogenEsch , and D. T. O'Hagan , “Aluminum Adjuvants—Back to the Future,” Pharmaceutics 15, no. 7 (2023): 1884, 10.3390/pharmaceutics15071884. 37514070 PMC10383759

[18] J. Zimmer , S. Vieths , and S. Kaul , “Standardization and Regulation of Allergen Products in the European Union,” Current Allergy and Asthma Reports 16, no. 3 (2016): 21, 10.1007/s11882-016-0599-4. 26874849

[19] J. Westdijk , B. Metz , N. Spruit , et al., “Antigenic Fingerprinting of Diphtheria Toxoid Adsorbed to Aluminium Phosphate,” Biologicals 47 (2017): 69–75, 10.1016/j.biologicals.2016.10.005. 28259519

[20] R. Riches‐Duit , L. Hassall , A. Kogelman , et al., “Characterisation of Tetanus Monoclonal Antibodies as a First Step Towards the Development of an In Vitro Vaccine Potency Immunoassay,” Biologicals 71 (2021): 31–41, 10.1016/j.biologicals.2021.04.002. 33910767

[21] A. Bullimore , T. Batten , S. Hewings , K. J. Weikersthal‐Drachenberg , and M. Skinner , “Cross‐Reactivity in Grasses: Biochemical Attributes Define Exemplar Relevance,” World Allergy Organization Journal 5, no. 10 (2012): 111–119, 10.1097/WOX.0b013e31826a10cf. 23282335 PMC3651171

[22] B. Fahlbusch , G. Schlenvoigt , W. D. Müller , B. Weber , and L. Jäger , “A Two‐Site Monoclonal Antibody ELISA for the Quantification of Group V Allergens in Grass Extracts,” Clinical and Experimental Allergy 24, no. 8 (1994): 752–757, 10.1111/j.1365-2222.1994.tb00986.x. 7982125

[23] EDQM , “Monograph on Diphtheria, Tetanus, Pertussis (Acellular Component) and Haemophilus Type B Conjugate Vaccine (Adsorbed) (07/2022:1932). European Pharmacopoeia 11.0”.

[24] A. Gray , A. R. M. Bradbury , A. Knappik , A. Plückthun , C. A. K. Borrebaeck , and S. Dübel , “Animal‐Free Alternatives and the Antibody Iceberg,” Nature Biotechnology 38, no. 11 (2020): 1234–1239, 10.1038/s41587-020-0687-9. 33046876

[25] B. Fahlbusch , W. D. Müller , G. Schlenvoigt , L. Jäger , R. Wahl , and B. Weber , “Monoclonal Antibody Immunoassay for Quantitative Analysis of Group V Allergens in Grass Pollen Extracts,” Clinical and Experimental Allergy 23, no. 9 (1993): 747–754, 10.1111/j.1365-2222.1993.tb00362.x. 10779305

[26] EDQM , “General Chapter 2.7.6. Assay of Diphtheria Vaccine (Adsorbed) (01/2008:20706) Corrected 6.0. European Pharmacopoeia 11.0”.

[27] “Directive 2010/63/EU of European Parliament and Council of the European Union of 22 September 2010 on the Protection of Animals Used for Scientific Purposes.OJ L 276, 20.10.2010, 33–79. 22 September 2010”.

[28] D. S. McVey , J. E. Galvin , and S. C. Olson , “A Review of the Effectiveness of Vaccine Potency Control Testing,” International Journal for Parasitology 33 (2003): 507–516, 10.1016/S0020-7519(03)00067-5. 12782051

