# Supplementary figures and images for: Immunological In Vitro Assay for Quantification of Adjuvanted Allergoids
Source: Allergy. 2025 Mar 31;80(7):2008–17. doi: 10.1111/all.16543 (PMC12261872; doi:10.1111/all.16543)

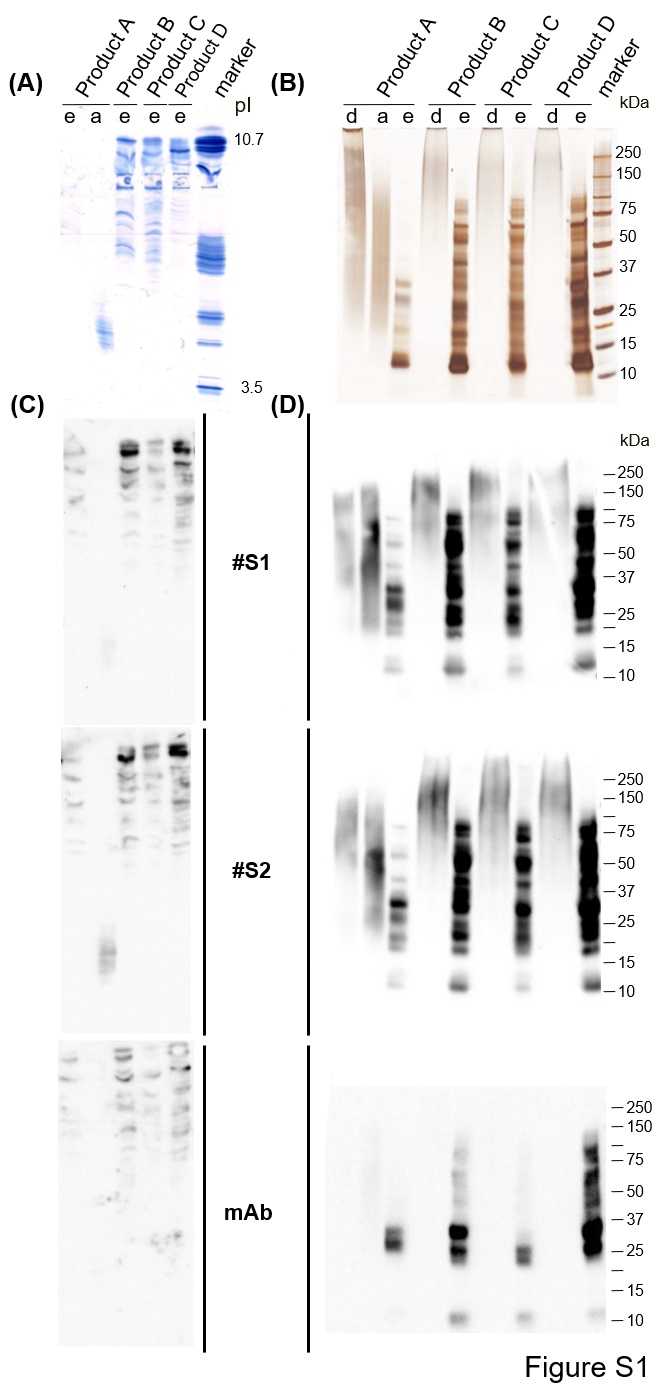

Supplement: Supplementary file 1 — Figure S1. [file ALL-80-2008-s002.jpg]
