# Supplementary material for: Immunological In Vitro Assay for Quantification of Adjuvanted Allergoids
Source: Allergy. 2025 Mar 31;80(7):2008–17. doi: 10.1111/all.16543 (PMC12261872; doi:10.1111/all.16543)
Supplement: Supplementary file 2 — Appendix S1. [file ALL-80-2008-s001.docx]

# **Supplementary Information**

**Immunological *in vitro* Assay for quantification of adjuvanted Allergoids**

S. Schlünder^1^, J. Echternach^2,#^ (ORCID.iD 0000-0003-2513-7487), D. Bartel^2^, V. Mahler^2^ (ORCID.iD 0000-0001-6471-1811), M.D. Mühlebach^1^ (ORCID.iD [0000-0002-7069-5018](https://orcid.org/0000-0002-7069-5018)), F. Führer^2^

^1^Veterinary Medicine Division, Paul-Ehrlich-Institut, Langen, Germany

^2^Allergology Division, Paul-Ehrlich-Institut, Langen, Germany

^#^present affiliation: Haematology, Cell and Gene Therapy Division, Paul-Ehrlich-Institut, Langen, Germany

# **Material and Methods**

Polyclonal Antibodies

Polyclonal antibodies (rabbit anti-grass pollen allergoid sera) were generated by immunizing Zika rabbits with a non-adjuvanted grass pollen allergoid intermediate manufacturing stage (product A) by a service provider for custom antibody production (BioGenes GmbH, Berlin, Germany). In short, pre-immune serum of two rabbits was drawn before (day 0) immunization by subcutaneous injections with grass pollen allergoid (250 μg protein resolved in Tris buffered saline and formulated with Freund's complete adjuvant (Sigma-Aldrich, Taufkirchen, Germany) in a 1:1 ratio). Seven booster immunizations on day 7, 14, 28, 56, 63, 84 and 91 following the first immunization were conducted, each containing 100 μg protein formulated in a 1:1 ratio in Freund's incomplete adjuvant (Sigma-Aldrich). Sera were collected at day 98 and stored at - 20 °C until use.

Allergen extract, allergoid and AIT drug product samples

All samples of AIT drug products as well as native allergen extracts and non-adjuvanted allergoids, so called pre- and intermediate manufacturing stages, used in this project were provided by the allergen manufacturers within the official batch release procedure. Their consent for use in this method development was obtained. The samples examined are presented in an anonymized form (grass pollen AIT-DPs A, B, C and D) due to the required neutrality and impartiality of the higher authority. AIT-DPs (aluminum hydroxide-adsorbed allergoids) of four different allergen manufacturers (Allergopharma GmbH & Co. KG (Reinbek, Germany), HAL Allergy B.V. (Leiden, Netherlands), LETI Pharma GmbH (Ismaning, Germany) and ROXALL Medizin GmbH (Oststeinbek / Hamburg, Germany)) were investigated. As there are no international reference materials for allergoids or adjuvanted allergoids available, one batch (batch 1) of a grass pollen AIT-DP A was defined as in house reference preparation (IHR). Native allergen extracts (from pollen of grass, rye (*Secale cereale*), wheat (*Triticum aestivum*), oat (*Avena sativa*), birch (*Betula verrucosa*), hazel (*Corylus avellana*), alder (*Alnus glutinosa*), pellitory (*Parietaria judaica*), mugwort (*Artemisia vulgaris*), plantain (*Plantago lanceolata*) and house dust mites (*Dermatophagoides farinae* and *Dermatophagoides pteronyssinus*)), as well as the cross-linked allergoids, both non-adjuvanted precursors of AIT drug products, were used in isoelectric focusing, sodium dodecyl sulfate polyacrylamide gel electrophoresis (SDS-PAGE) and immunoblotting analysis. The samples were stored at 2 °C to 8 °C or -15 ° to -25 °C as indicated by the respective manufacturer. Protein content of the samples was quantified by elemental nitrogen analysis according to the Kjeldahl method described in *European Pharmacopoeia* 11.0 (2.5.33, 7B method)^1^. For analysis of grass pollen AIT-DP stability, batch 1 of grass pollen AIT-DP A was subjected to a forced degradation by heating the sample to 95°C for 5 min. This heat stressed sample was cooled down at room temperature (RT) and immediately analyzed in comparison to an untreated control sample with allergoid content assay (ACA).

Desorption of AIT-DPs

Desorption protocol was performed according to *Pharm Eur*. 07/2022:1932^2^, one batch of each AIT-DPs A-D was collected by centrifugation of 6 mL at 13,000 g for 3 min. Supernatant was discarded and pellets were dissolved in 1 mL desorption buffer (10 % sodium citrate (w/v)). Desorption preparations were incubated at 37°C and 30 rpm on an overhead-rotator overnight. Remaining aluminum hydroxide particles were sedimented by centrifugation at 2,000 g for 2 minutes. Supernatant contains the desorbed allergoides and was subjected to further investigation.

Isoelectric focusing (IEF) Coomassie staining

Native allergen extracts and cross-linked allergoids, both non-adjuvanted precursors of AIT drug products, of allergen sources listed above (50 μg per lane in 6 M urea and IEF sample buffer (SERVA, Heidelberg, Germany)) were separated on self-casted thin layer 5 % IEF PA gels (6 M Urea, 3 % Servalyt™ 3-6 carrier ampholytes (w/v), 0.1 % Ammonium persulfate (w/v) and 0.1 % Tetramethylethylenediamine (v/v) (SERVA)) at a constant current of 12 mA per gel according a protocol adapted to Lee et al. 2001^3^ with minor changes. IEF marker 3-10 (SERVA) was used as standard for calibration of the isoelectric point (pI). After isoelectric focusing, IEF PA gels were stained with Coomassie brilliant blue staining (SERVA) according to manufacturer’s procedures.

SDS-PAGE

SDS-PAGE was performed to analyze the protein profile of native allergen extracts and cross-linked allergoids, both non-adjuvanted precursors of AIT drug products, of allergen sources listed above. Gels were loaded with 5 μg protein per lane in urea buffer^4^ after denaturation at 95 °C for 10 min. Electrophoresis was performed with NuPAGE 4-12 % Bis-Tris Gels (Invitrogen, Thermo Fisher Scientific, Dreieich, Germany) under reducing conditions according to manufacturer´s instructions. Precision Plus Protein™ Unstained Protein (Bio-Rad, Feldkirchen, Germany) was used as molecular weight marker. Proteins were directly visualized by a silver staining protocol adapted from Blum et al. 1987^5^ with minor changes. Briefly, after fixation of the gels in 30 % ethanol (v/v), 10 % acetic acid (v/v) for at least 30 min at RT, gels were incubated for at least 30 min in sodium thiosulfate solution (30 % ethanol (v/v), 0.8 M sodium acetate, 0.5 % glutaraldehyde (v/v), 20 mM sodium thiosulfate). After rinsing the gels three times with water, impregnation with silver nitrate solution (5.9 mM silver nitrate, 0.02 % formaldehyde (v/v)) is performed for 15-60 min under light protection. Gels were incubated in developer solution (0.24 M sodium carbonate, 0.01 % formaldehyde (v/v)) for a few seconds to a maximum of 5 min (depending on the desired intensity of the bands) and transferred to 50 mM EDTA solution to stop reduction of silver ions. Before scanning, the gels were rinsed three times with water.

Immunoblotting

For IEF immunoblotting, separated samples on IEF PA gels with NetFix™ (SERVA) were transferred (100 V, 200 mA, 10 W, 90 min) with a Semi-Dry Blotting Buffer Kit (SERVA) to 0.2 μm nitrocellulose membranes (Merck, Darmstadt, Germany) according to manufacturer´s instructions. Western immunoblotting was performed using a tank-blotting system (Mini Trans-Blot Cell (Bio-Rad)) to transfer the proteins to 0.2 μm nitrocellulose membranes (Merck). Immune detection steps were identical for IEF and Western immunoblotting. The membranes were blocked in phosphate-buffered saline containing 0.05 % Tween-20 (PBS-T) and 2 % milk powder (Carl Roth, Karlsruhe, Germany) (blocking buffer) for at least 1 h. After washing in PBS-T (4 x 4 min), the membranes were incubated with rabbit anti-grass pollen allergoid sera (1:100,000) or mouse anti-Phl p 5 monoclonal antibody (mAb) 1D11 (InBio, Cardiff, UK) (1:10,000) diluted in blocking buffer for at least 1 h at RT with gentle rocking. After washing with PBS-T (4 x 4 min), the membranes were further incubated for 1 h at RT with AffiniPure Goat Anti-Rabbit IgG, HRP (Jackson ImmunoResearch Laboratories, Cambridgeshire, UK) (1:100,000) (secondary antibody for anti-grass pollen allergoid sera) or AffiniPure Goat Anti-Mouse IgG, HRP (Jackson ImmunoResearch Laboratories) (1:100,000) (secondary antibody for anti-Phl p 5 mAb). Following further washes with PBS-T (4 x 4 min), peroxidase activity was visualized with the ECL™ Prime Western-Blot-System (Merck) according to manufacturer´s instructions and a Lumi-Imager F1 Imaging System (Roche, Basel, Switzerland).

Data analysis

Relative allergoid content of each adjuvanted allergoid AIT drug product against in-house reference was calculated by parallel line or sigmoid analysis using the software CombiStats^TM^ (EDQM, Strasbourg, France). Each assay was checked for validity with respect to regression, parallelism, and linearity of the curves. Graphs, column charts and statistical analysis were generated/performed with GraphPad Prism 9.0 (GraphPad Software, Boston, MA.). For data validation, significance analysis was performed as one sample t-test for RAC analysis in FIGURE 3 B, 4 B and 6 B with three independent experiments, each. One sample Wilcoxon test was applied for RAC analysis in FIGURE 5 B due to at least five independent experiments performed. Limit of detection (LoD) was determined by three-fold SD of the aluminum control signal at the highest dilution (1:128) according ICH Topic Q2 (R2) (Validation of Analytical Procedures)^6^.

References

1. EDQM. General Chapter 2.5.33. Total Protein (01/2008:20533). European Pharmacopoeia 11.0.

2. EDQM. Monograph on Diphtheria, Tetanus, Pertussis (Acellular Component) and Haemophilus Type B Conjugate Vaccine (Adsorbed) (07/2022:1932). European Pharmacopoeia 11.0.

3. Lee, SS, Lawton, JW, Ko, KH, Lam, KM, Lin, CK. alpha-1 antitrypsin phenotypes by isoelectric focusing in a metropolitan southern Chinese population. Journal of clinical pathology 2001;54:798–800.

4. Plemper, RK, Hammond, AL, Cattaneo, R. Measles virus envelope glycoproteins hetero-oligomerize in the endoplasmic reticulum. The Journal of biological chemistry 2001;276:44239–46.

5. Blum, H, Beier, H, Gross, HJ. Improved silver staining of plant proteins, RNA and DNA in polyacrylamide gels. Electrophoresis 1987;8:93–99.

6. European Medicines Agency. Committee for Medicinal Products for Human Use (CHMP): ICH Q2(R2) Guideline on validation of analytical procedures (EMA/CHMP/ICH/82072/2006).

**Figure Legends**

FIGURE S1. Specificity determination of primary antibodies. Representative result testing non-adjuvanted allergen extracts (e) non-adjuvanted allergoids (a) and desorbed allergoids (after desorption process) (d) from manufacturer A-D (A) IEF gel after Coomassie staining. (B) SDS-PAGE after silver staining. (C) and (D) immunoblotting with rabbit anti-grass pollen allergoid serum 1 (#S1), rabbit anti-grass pollen allergoid serum 2 (#S2), anti-Phl p 5 mAb (mAb).
